# Re-evaluation of Diadenosine Tetraphosphate (Ap_4_A) From a Stress Metabolite to *Bona Fide* Secondary Messenger

**DOI:** 10.3389/fmolb.2020.606807

**Published:** 2020-11-17

**Authors:** Freya Ferguson, Alexander G. McLennan, Michael D. Urbaniak, Nigel J. Jones, Nikki A. Copeland

**Affiliations:** ^1^Biomedical and Life Sciences, Faculty of Health and Medicine, Lancaster University, Lancaster, United Kingdom; ^2^Materials Science Institute, Lancaster University, Lancaster, United Kingdom; ^3^Department of Molecular Physiology and Cell Signalling, Institute of Systems, Molecular and Integrative Biology, University of Liverpool, Liverpool, United Kingdom

**Keywords:** Ap_4_A, diadenosine, nucleotide signaling, DNA replication and genotoxic stress, mRNA caps, cGAS/STING, MITF

## Abstract

Cellular homeostasis requires adaption to environmental stress. In response to various environmental and genotoxic stresses, all cells produce dinucleoside polyphosphates (Np_n_Ns), the best studied of which is diadenosine tetraphosphate (Ap_4_A). Despite intensive investigation, the precise biological roles of these molecules have remained elusive. However, recent studies have elucidated distinct and specific signaling mechanisms for these nucleotides in prokaryotes and eukaryotes. This review summarizes these key discoveries and describes the mechanisms of Ap_4_A and Ap_4_N synthesis, the mediators of the cellular responses to increased intracellular levels of these molecules and the hydrolytic mechanisms required to maintain low levels in the absence of stress. The intracellular responses to dinucleotide accumulation are evaluated in the context of the “friend” and “foe” scenarios. The “friend (or alarmone) hypothesis” suggests that Ap_n_N act as *bona fide* secondary messengers mediating responses to stress. In contrast, the “foe” hypothesis proposes that Ap_n_N and other Np_n_N are produced by non-canonical enzymatic synthesis as a result of physiological and environmental stress in critically damaged cells but do not actively regulate mitigating signaling pathways. In addition, we will discuss potential target proteins, and critically assess new evidence supporting roles for Ap_n_N in the regulation of gene expression, immune responses, DNA replication and DNA repair. The recent advances in the field have generated great interest as they have for the first time revealed some of the molecular mechanisms that mediate cellular responses to Ap_n_N. Finally, areas for future research are discussed with possible but unproven roles for intracellular Ap_n_N to encourage further research into the signaling networks that are regulated by these nucleotides.

## Introduction

**FIGURE 1:**
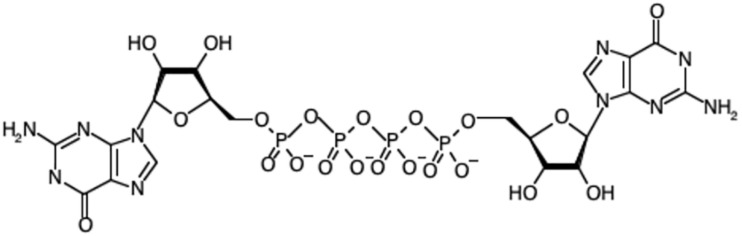
The chemical structure of diadenosine tetraphosphate (Ap_4_A).

Diadenosine polyphosphates (Ap_n_As) are a class of nucleotide found in prokaryotes and eukaryotes (Kisselev et al., 1998; McLennan, 2000). They consist of two adenosine moieties linked by a polyphosphate chain containing typically 3–6 phosphates attached via phosphoester bonds to the respective 5′-OH groups (Figure 1). Since their initial discovery, roles for diadenosine polyphosphates (Ap_n_A) and other dinucleoside polyphosphate species (Np_n_N, where N is uridine, cytosine, guanine or adenine and *n* is 3–6), have remained elusive in all Kingdoms of life. There has been debate surrounding the roles of these molecules for decades. However, their functional characterization has been limited due to the complexity of identifying specific interaction partners and this has led to the alternative proposal that Ap_4_A and other Np_n_N may simply be damage metabolites rather than intracellular signaling molecules. Dinucleoside polyphosphates are generated in response to stress, consistent with a stress-related function. Consequently, interest has mainly focused on trying to establish Np_n_N, particularly Ap_4_A and Ap_4_N, as signaling molecules, and in particular stress alarmones. The extracellular roles of Ap_4_N that includes their roles as extracellular messengers in the nervous, ocular and cardiovascular systems through activation of purinoceptors has been covered in detail elsewhere and will not be considered here (Jankowski et al., 2009).

### Ap_n_Ns Are Synthesized in Response to Physiological, Environmental, and Genotoxic Stress

Bacteria, protists, yeasts, invertebrates and mammals have mechanisms for the synthesis and hydrolysis of Ap_n_N. The balance between synthesis and hydrolysis is required to maintain Ap_n_N at a low level under normal conditions. However, Ap_n_N levels increase in response to certain types of stress throughout all these domains of life. For example, in *Salmonella typhimurium* Ap_4_N levels increase in response to certain specific oxidative stresses, with Ap_4_A reaching a maximal concentration of 365 μM after 30 min of CdCl_2_ treatment compared to concentrations of <3 μM in cells undergoing non-oxidative stress (Bochner et al., 1984). Additionally, hyperthermic treatment is associated with membrane damage (Lepock, 1982) and this kind of treatment also increases dinucleoside polyphosphate levels. Increasing the temperature of *S. typhimurium* cells from 28 to 50°C resulted in an approximately 10-fold increase in Ap_4_A and Ap_4_G after 5 min, which then continued to increase (Lee et al., 1983). These early results were instrumental in the formulation of the alarmone hypothesis that Ap_4_A was an intracellular signal of molecular stress that orchestrated subsequent recovery mechanisms (Varshavsky, 1983). An increase in Ap_4_A and Ap_4_G levels was also identified in *Synechococcus* sp. strain PCC 6301 exposed to 50°C, however, this increase was lower than that of Ap_3_A and Ap_3_G (Palfi et al., 1991). In both *S. typhimurium* and *Synechococcus*, the increase in nucleotides was dependent on the severity of the temperature change (Lee et al., 1983; Palfi et al., 1991). Treatment of *S. typhimurium* with 10% ethanol also resulted in an increase in Ap_4_A with levels rising from <5 μM to >50 μM over 50 min, but with less of an effect on Ap_4_G (Lee et al., 1983). In contrast, treatment of *Synechococcus* with 10% ethanol produced no effect, while treatment with heavy metal ions induced Ap_4_A accumulation to different extents dependent on the metal (Palfi et al., 1991). Ap_4_N levels in *Saccharomyces cerevisiae* also increased after treatment with cadmium acetate (Baltzinger et al., 1986). In *Drosophila* cells concentrations of 1 mM CdCl_2_ and above increased these nucleotides by over 100-fold, to 30 μM Ap_4_A and 39 μM Ap_4_G after 6 h of treatment (Brevet et al., 1985).

Similarly, oxidative stress induced by 0.1 mM dinitrophenol increased levels of Ap_4_A and Ap_4_G in the slime mold *Physarum polycephalum* by 3- to 7-fold (Garrison et al., 1986). Hypoxia also increased Ap_4_A and Ap_4_G levels by 6- to 7-fold within 40 min in *P. polycephalum*, an increase that rapidly declined in normoxic conditions (Garrison et al., 1989). Importantly, Ap_4_A levels in *Escherichia coli* increase 20-fold after kanamycin treatment, which generates hydroxyl radicals leading to oxidative stress (Ji et al., 2019). Aminoglycosides cause bacterial cell death by binding prokaryotic ribosomes and causing mistranslation of proteins that accumulate at the bacterial membrane, which promotes membrane permeabilization (Busse et al., 1992; Mingeot-Leclercq et al., 1999). The rise in Ap_4_A levels upon kanamycin treatment increases the effectiveness of aminoglycoside-induced bacterial cell death. As the use of aminoglycosides can be limited by their toxicity, combined therapy with an agent that increases intracellular Ap_4_A may offer a means to improve the potency of aminoglycosides at lower doses (Ji et al., 2019).

Thermal shock also promotes accumulation of Ap_4_A in yeast, plants and mammalian cells. Single cell eukaryotes such as *S. cerevisiae* demonstrated a response to heat shock at 46°C with basal Ap_4_N levels of approximately 0.08 μM increasing 50-fold (Baltzinger et al., 1986). However, the authors concluded that such increases were always associated with irreversible processes leading to cell death, thus giving early support to the damage metabolite hypothesis. Heat-shock from 19 to 37°C also induced a 2.2 to 3.3-fold increase in Ap_4_A and other Ap_n_N in *Drosophila* cells (Brevet et al., 1985). Thermal stress also increases Ap_4_A levels in mammalian cells. Simian virus 40-transformed mouse 3T3 mammalian cells exposed to hyperthermic treatment for 30 min produced elevated Ap_n_N levels 1 h after treatment, >90% of which were shown to be Ap_4_A (Baker and Jacobson, 1986). Ethanol, cadmium and arsenite treatment also increased Ap_n_N (Baker and Jacobson, 1986). Again, however, evidence was also presented that significant heat-induced Ap_4_N increases in *Xenopus* oocytes and HTC hepatoma cells are associated with cell death (Guédon et al., 1986).

There is also considerable evidence that Ap_4_N are produced is response to genotoxic stress, including to agents causing DNA single-stranded breaks, although not necessarily at physiologically relevant doses. For example, *N*-methyl-*N*′-nitro-*N*-nitrosoguanidine (MNNG), bleomycin, nitroquinoline-1-oxide (4NQO) and UV irradiation all increased Ap_4_N several-fold in human fibroblasts. Co-administration of cytosine arabinoside with 4NQO and UV irradiation significantly increased their otherwise modest effect by inhibiting nucleotide excision repair and causing subsequent replication stress (Baker and Ames, 1988). Similar observations were made with MNNG in HTC hepatoma cells with the increase in Ap_4_N being greater and more prolonged in the presence of the PARP inhibitor 3-aminobenzamide (Gilson et al., 1988). Inter-strand crosslinking agents are highly effective and unlike many other types of genotoxic agents, induce Ap_4_A at physiologically relevant doses. Mitomycin C at a level that caused no growth inhibition (100 nM) increased Ap_4_A levels 7 to 8-fold in HeLa and Chinese hamster AA8 cells, and 9-fold in mouse embryonic fibroblasts (MEFs) while 10 μM 1,2,3,4-diepoxybutane caused a 3-fold increase in AA8 cells (Marriott et al., 2015). Cells depleted of certain DNA repair proteins including XRCC1, aprataxin, PARP1 and FANCG also showed increases in Ap_4_N up to 14-fold with mitomycin C treatment enhancing these increases several-fold more. All these observations are indicative of the increases in Ap_4_N being mediated by DNA damage that prevents replication fork progression, such as that efficiently induced by ICL agents. Intriguingly, inactivation of the NUDT2 Ap_4_A hydrolase in KBM-7 CML cells, that leads to a 175-fold increase in intracellular Ap_4_A (Marriott et al., 2016). Interestingly, a significant portion of the damage-induced Ap_4_A in AA8 cells and its XRCC1-deficient EM9 derivative by mitomycin C was mono- and di-ADP-ribosylated. No increase in diadenosine triphosphate (Ap_3_A) was found. Cells treated in this way showed near full viability, indicating that the increases in Ap_4_N were not due to irreversible cell death, but could be a functional response to genotoxic damage (Marriott et al., 2015). Regardless of the severity of stress required to elicit increased levels of Ap_4_N, stress-induced increases do not by themselves indicate a true alarmone or secondary messenger function. Ideally, signaling molecules should have specific and regulated mechanisms of synthesis and degradation. The synthesis of the signal molecule should increase in response to stimuli, leading to interactions with specific downstream targets such as transcription factors or allosteric binding proteins that mediate a cellular response to the stress. Importantly, the effect of the signaling molecules should be reversible once the stimulus ceases. Each of these criteria are met for Ap_4_N and Ap_4_A in specific contexts and will be explored further below.

### Mechanisms of Ap_4_N Synthesis

Ap_4_Ns are synthesized by non-canonical activities of certain enzymes that often involve an acyl-adenylate and/or enzyme-adenylate intermediate, and this non-canonical activity is often enhanced during stress responses (Table 1). Ap_4_A was first discovered in the 1960s as a product of the reaction between ATP and lysyl-tRNA synthetase (LysRS) in the presence of L-lysine (Zamecnik et al., 1966). Ap_4_A and other Ap_n_Ns were later found to be synthesized by several members of the aminoacyl-tRNA synthetase family in a reversible process involving synthesis of an aminoacyladenylate intermediate followed by reaction with an acceptor such as ATP or NTP/NDP (Goerlich et al., 1982; Brevet et al., 1989). Aminoacyl-tRNA synthetase-mediated production of Ap_4_A occurs both during normal cell growth and at times of stress, and for certain synthetases is stimulated by Zn^2+^ ions (Plateau and Blanquet, 1982; Brevet et al., 1989). Later an additional amino acid-independent mechanism of Ap_4_A synthesis involving the direct condensation of two ATPs by glycyl-tRNA synthetase (GlyRS) was identified. In this mechanism the first ATP binds to a site conserved in all class II tRNA synthetases while the second ATP binding pocket is specific to the GlyRS (Guo et al., 2009).

**TABLE 1:** Enzymes synthesizing dinucleoside polyphosphates.

Enzyme	Source*^a^*	Products*^b^*	References
Aminoacyl-tRNA synthetases	Eukaryotes, prokaryotes	Ap_3__–__5_N, dAp_4_dA, Ap_3_Gp_2_	Goerlich et al., 1982; Brevet et al., 1989; Guo et al., 2009
Firefly luciferase	*Photinus pyralis*	Ap_3__–__5_N, Ap_3__–__5_dN, Gp_4_G	Guranowski et al., 1990; Sillero and Sillero, 2000
DNA ligase	T4 phage, *Homo sapiens* (Lig3)	Ap_3_A, Ap_4_A, Ap_4_G, Ap_4_dA	Madrid et al., 1998; McLennan, 2000; Sillero and Sillero, 2000
RNA ligase	T4 phage	Ap_4_A, Ap_4_G, Ap_4_dG, Ap_4_C, Ap_4_dC, Ap_3_A	Atencia et al., 1999; Sillero and Sillero, 2000
Acyl-coenzyme A synthetase	*P. fragi*	Ap_4__–__6_A, Ap_4_N	Fontes et al., 1998
4-coumarate: coenzyme A ligase	*A. thaliana*	Ap_4_A, Ap_5_A, dAp_4_dA	Pietrowska-Borek et al., 2003
UTP:glucose-1-phosphate uridylyl transferase	*S. cerevisiae*	Up_4_N, Up_5_A, Up_5_G	Guranowski et al., 2004
Ap_4_A phosphorylase	*S. cerevisiae*	Ap_4_N, Ap_4_dA	Brevet et al., 1987; Guranowski et al., 1988
GTP:GTP guanylyltransferase	*A. franciscana*	Gp_4_G, Gp_4_A, Gp_3_G, Gp_3_A	Liu and McLennan, 1994
GTP:mRNA guanylyltransferase	*S. cerevisiae*	Gp_4_N, Gp_3_N	Wang and Shatkin, 1984
Non-ribosomal peptide synthetase	*B. brevis, E. coli*	Ap_4_A, Ap_5_A, Ap_6_A	Dieckmann et al., 2001; Sikora et al., 2009
Ubiquitin, SUMO and NEDD8-activating enzymes	*H. sapiens*	Ap_4_A, Ap_3_A	Götz et al., 2019
Vascular endothelial growth factor receptor 2	*H. sapiens*	Ap_2_A, Ap_4_A, Ap_6_A, Ap_3_G, Ap_4_U, Gp_2_G, Up_4_U	Jankowski et al., 2013
Reverse transcriptase	HIV type 1	Ap_3_ddA, Ap_4_ddA, Gp_4_ddA*^c^*	Smith et al., 2005

*^a^Orthologous activities may also be present in related organisms. ^b^Examples of products that have been detected; the list is not exhaustive and may vary with source of enzyme; only products likely to occur naturally are shown. ^c^Although dideoxynucleotides are not natural, their formation indicates the potential for natural dinucleotide synthesis by reverse transcriptases.*

Firefly luciferase and several other ligases including acyl-coenzyme A synthetase, T4 DNA ligase, DNA ligase III and T4 RNA ligase are also capable of synthesizing Ap_4_A and other Ap_4_Ns (McLennan, 2000; Sillero and Sillero, 2000; Fraga and Fontes, 2011). The mechanism behind firefly luciferase-mediated synthesis of Ap_4_N involves initial production of an enzyme-luciferyl-AMP intermediate (Guranowski et al., 1990). Similarly, acyl-CoA synthetase from *Pseudomonas fragi* can synthesize Ap_4_N possibly via an acyl-CoA-AMP intermediate (Fontes et al., 1998) and 4-coumarate:CoA ligase via a cinnamoyl-CoA intermediate (Pietrowska-Borek et al., 2003). An acyl-AMP intermediate is not essential as T4 RNA ligase can catalyze Ap_4_N synthesis through formation of a T4 RNA ligase-AMP complex, where ATP donates the AMP, followed by an NTP acting as an acceptor for the AMP directly from the ligase-AMP (Atencia et al., 1999). Different rates of synthesis can be seen for the different Ap_4_Ns, decreasing in the order Ap_4_dG > Ap_4_G > Ap_4_dC > Ap_4_C (Atencia et al., 1999). Similarly, T4 DNA ligase is able to synthesize Ap_4_A and Ap_4_G among other Np_n_N by formation of the ligase-AMP intermediate followed by reaction of NTP with the AMP (Madrid et al., 1998). In the context of genotoxic stress in mammalian cells, Ap_4_A synthesis may be mediated by the repair enzyme DNA ligase III (McLennan, 2000; Marriott et al., 2015).

There are also Ap_4_N synthetases that may operate specifically under stress conditions. For example, the heat-inducible *E. coli* LysU is a particularly efficient synthesizer of Ap_n_N (Chen et al., 2013). The *Bacillus brevis* non-ribosomal peptide synthetase that responds to nutritional stress can also synthesize Ap_4_A (Dieckmann et al., 2001) as can the EntE subunit of the analogous complex from *E. coli* during iron starvation via an adenylated aryl-acid intermediate (Sikora et al., 2009). More recently, ubiquitin and ubiquitin-like E1 activating enzymes have also been shown to synthesize Ap_4_A as a side reaction in the ubiquitin and ubiquitin-like activation pathway (Götz et al., 2019). The ubiquitin activating enzyme UBA1 can initiate synthesis of Ap_4_A in a mechanism involving an adenylate-UBA1 intermediate in a process that is inhibited by increased concentrations of the E2 ubiquitin-conjugating enzymes (Götz et al., 2019). In addition, ubiquitin-like enzymes NEDD8- and SUMO-activating enzymes are also able to synthesize Ap_4_A, but with lower activity (Götz et al., 2019). Thus, the cellular responses to stress promote the synthesis and accumulation of Ap_4_A and other Ap_n_N species by degenerate mechanisms. While the wide range of enzymes able to synthesize Ap_n_N *in vitro* may be seen as an argument against specific signaling roles for Ap_n_N, the degenerate mechanisms ensure the timely and rapid accumulation of Ap_4_N in response to stress.

### Regulation of Intracellular Ap_4_N Levels by Regulated Synthesis and Hydrolysis

To effectively function as a signaling molecule, Ap_4_N levels must be precisely controlled to prevent inappropriate activity in the absence of physiological, environmental, oxidative or genotoxic stress. Since Ap_4_N are synthesized continuously even in unstressed cells, Ap_4_N are efficiently degraded within cells preventing their accumulation to toxic levels. Any Ap_4_N-mediated signaling function would also require such regulation and several families of Ap_4_N hydrolyzing enzymes have been found in prokaryotes and eukaryotes (Table 2). Hydrolysis of Ap_4_N can occur either symmetrically or asymmetrically. In *E. coli*, the major hydrolytic activity, bis(5′-nucleosyl)-tetraphosphatase (ApaH), cleaves Ap_4_A symmetrically yielding ADP and this activity is also present in other *β*- and *γ*- proteobacteria (Guranowski et al., 1983). Significantly, Ap_4_A levels increase by up to 100-fold in *ApaH*^–^ *E. coli* cells while a ∼10-fold decrease results from overexpression of the hydrolase, demonstrating the importance of ApaH for the regulation of Ap_4_A levels (Mechulam et al., 1985; Plateau et al., 1987; Farr et al., 1989). In Gram positive bacteria which lack the ApaH family enzyme, such as *Bacillus subtilis* and *Staphylococcus aureus*, the YqeK protein family of Ap_4_A hydrolases has recently been found. The YqeK family has a similar Ap_4_A cleavage specificity to the ApaH hydrolases and deletion of the *YqeK* gene from *B. subtilis* leads to increases in Ap_4_N content (Minazzato et al., 2020).

**TABLE 2:** Enzymes degrading dinucleoside polyphosphates.

Enzyme	Source/gene	Reaction*^a^*	References
“*Symmetrical*” Ap_4_A hydrolase	β- and γ-proteo bacteria (*ApaH*)	Ap_4_A → ADP + ADP	Guranowski et al., 1983; Guranowski, 2000
	Gram-positive bacteria (*YqeK*)	Ap_4_A → ADP + ADP	Minazzato et al., 2020
	*Physarum polycephalum*	Ap_4_A → ADP + ADP	Garrison et al., 1982
“*Asymmetrical*” Nudix Ap_4_A hydrolase	Animals (e.g., *Nudt2*), plants (e.g., *AtNUDT25*), proteobacteria (*RppH/NudH/IalA/YgdP*), mycobacteria (*MutT1*)	Ap_4_A → AMP + ATP	Guranowski, 2000; McLennan, 2006; Kraszewska, 2008; Arif et al., 2017
Ap_4_A phosphorylase	*S. cerevisiae* (*Apa1, Apa2*), protists, cyanobacteria, mycobacteria (*MtApa*)	Ap_4_A + Pi ↔ ADP + ATP	Guranowski and Blanquet, 1985; McLennan et al., 1994, 1996; Hou et al., 2013; Honda et al., 2015
HIT family hydrolases	*S. cerevisiae* (*Aph*), *H. sapiens* (*FHIT*)	Ap_3_A → AMP + ADP	Barnes et al., 1996
	*Schizosaccharomyces pombe* (*Aph1*)	Ap_4_A → AMP + ATP	Ingram and Barnes, 2000
Non-specific phosphodi esterases	*H. sapiens* (e.g., *NPP1*, *NPP3, NPP4*)	Ap_4_A → AMP + ATP	Guranowski, 2000

*^a^The reaction with the best characterized or favored substrate is shown; other substrates may also be effective.*

Asymmetrically cleaving Ap_4_N hydrolases, that yield ATP and AMP as products of Ap_4_A hydrolysis, fall into two families: the NUDIX family and the HIT family of dinucleotide hydrolases. The NUDIX superfamily is conserved from bacteria to man. NUDIX family Ap_n_N hydrolases are found in Gram-negative bacteria including *Bartonella bacilliformis* (IaIA) (Cartwright et al., 1999; Conyers and Bessman, 1999), *E. coli* (variously known as NudH, YgdP, IalA and RppH) (Bessman et al., 2001) and the nudix family paralog MutT1 in *Mycobacteria* (Arif et al., 2017). Quantitatively, YgdP is less important in controlling the intracellular levels of Ap_4_A than ApaH in *S. typhimurium* (Ismail et al., 2003). However, both RppH and ApaH have the additional function of degrading both capped and uncapped 5′ ends of bacterial mRNAs. The major Ap_4_N hydrolase is encoded in humans by the *NUDT2* gene (Thorne et al., 1995; McLennan, 2006) and deletion of *NUDT2* results in a 175-fold increase in intracellular Ap_4_A (Marriott et al., 2015, 2016) confirming its significance as a major Ap_4_A hydrolase.

*Schizosaccharomyces pombe* lacks a nudix Ap_4_N hydrolase, and utilizes a HIT family enzyme Aph1 to cleave Ap_4_N asymmetrically (Huang et al., 1995). Aph1 contains the catalytic HIT motif (HφHφHφφ) and is a homolog of the human tumor suppressor FHIT protein, which prefers Ap_3_N substrates (Barnes et al., 1996). *Saccharomyces cerevisiae* also lacks an appropriate Nudix hydrolase but instead has two Ap_4_A phosphorylases, Apa1 and Apa2, that share 60% identity and which display phosphorolytic activity toward Ap_4_A and other Np_n_Ns, yielding NTP and NDP (Guranowski and Blanquet, 1985; Plateau et al., 1990; Hou et al., 2013). These phosphorylases are found in many protists (McLennan et al., 1994), and can both hydrolyze and synthesize Np_n_Ns including the non-adenylated dinucleotides. They are similar to the GalT proteins of the HIT family, which have a typical HXHXQφφ motif. Cyanobacteria and mycobacteria also have Ap_4_A phosphorylases (McLennan et al., 1996; Mori et al., 2010; Honda et al., 2015). The enzyme from *M. tuberculosis* is unusual in that it has a proper HIT motif (HφHφHφφ) more similar to the HIT family hydrolases than other Ap_4_A phosphorylases, but other amino acid changes in the active site may account for its phosphorylase activity (Mori et al., 2011). Members of the relatively non-specific nucleotide pyrophosphatase/phosphodiesterase (NPP) family can also hydrolyze Np_n_N but their relevance to the regulation of intracellular dinucleotide levels is not clear (Guranowski, 2000).

### Ap_4_N-Mediated Regulation of mRNA Stability and Gene Expression in Bacteria

**FIGURE 2:**
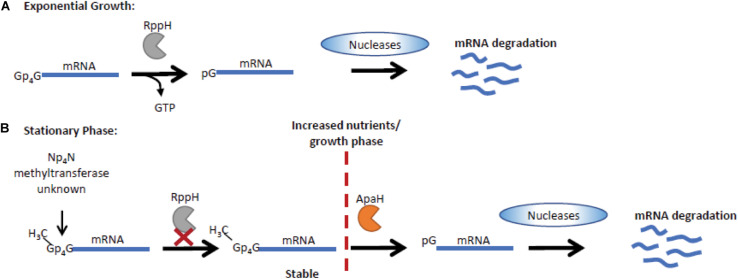
Np_4_N regulates transcript stability in stationary phase in *E. coli.* **(A)** Np_4_N caps can be incorporated into mRNA during transcription initiation. During exponential growth, Np_4_N caps can be removed by RppH, generating an NTP and a 5′ p-terminus on the mRNA, which is then degraded by the RNase E endonuclease (Luciano et al., 2019). **(B)** In stationary phase, methylation of the Np_4_N caps increases their stability as RppH is unable to process methyl-Np_4_N-caps. The processing of methyl-Np_4_N-caps is mediated by ApaH that is downregulated during stationary phase, increasing methyl-Np_4_N-mRNA stability (Hudeček et al., 2020). Under conditions where growth can resume, ApaH is activated, promoting cleavage of methylated caps, resulting in mRNA with a 5′ p-terminus that is efficiently degraded by RNase E nucleases.

The long-held notion that all bacterial mRNAs retain the 5′-triphosphate of the initiating nucleotide has now been displaced by the discovery of various 5′ cap structures that alter mRNA stability and contribute to regulation of gene expression. In *E. coli* 10–15% of mRNAs are capped by derivatives of NAD and CoA (Chen et al., 2009; Kowtoniuk et al., 2009). Importantly, Ap_n_Ns (where N can be A, C, G, or U and *n* = 3 to 5) are also used in *E. coli* as 5′-mRNA caps in response to oxidative stress. Increases in disulfide stress following CdCl_2_ treatment promotes inactivation of the ApaH hydrolase leading to an increase in intracellular Ap_n_N levels and accumulation of Np_n_ caps on the majority of mRNA transcripts. The novel Np_n_ caps extend mRNA half-life and potentially regulate gene expression (Luciano et al., 2019). The Np_n_ caps can arise either by direct addition of AMP from an aminoacyladenylate to an mRNA 5′-ppp terminus catalyzed by LysU or by utilization of preformed Ap_n_N as the initiating nucleotide by RNA polymerase. The strong influence on capping efficiency of changes to the untranscribed regions upstream of the promoter, particularly at position -1, suggests that the latter mechanism predominates (Luciano and Belasco, 2020). Ap_4_Ns can be incorporated at transcription initiation much more efficiently than ATP or Ap_n_Ns with other phosphate chain lengths, which demonstrates specificity in the interaction between Ap_4_N and the RNA polymerase/DNA template. Furthermore, the nature of the nucleotide at the +1 position differentially affects which Ap_4_N is incorporated with all four used when +1 is T but only Ap_4_G, Ap_4_C and Ap_4_U when +1 is C, G or A, respectively (Luciano and Belasco, 2020). Subsequently it was demonstrated that both bacteriophage T7 RNA polymerase and *E. coli* RNA polymerase are capable of capping mRNA transcripts *in vitro* (Hudeček et al., 2020). In addition, Ap_n_N-caps can be formed using other dinucleoside polyphosphates including Ap_3_A, Ap_3_G, and Ap_5_A. An additional layer of regulation is mediated by methylation of Ap_4_N molecules by an unknown methyltransferase. Methylation of Ap_4_N is associated with growth phase. Their abundance is low during exponential growth and increased during stationary phase when m^6^Ap_3_A, m^7^Gp_4_Gm, mAp_5_G, mAp_4_G, mAp_5_A, and 2mAp_5_G could be detected (Hudeček et al., 2020). The additional regulation of mRNA stability by methylation of the dinucleoside caps has important implications for mRNA stability and gene expression during stationary phase (Figure 2).

Both ApaH and RppH are capable of catalyzing Ap_4_N cap removal from mRNA *in vitro*. ApaH hydrolyzes the Np_4_ cap yielding an NDP and a 5′-pp terminus while, with lower efficiency, RppH generates a 5′-p terminus and NTP. However, *in vivo* in it seems likely that they act in concert with RppH, already established as acting upon 5′-ppp and 5′-pp termini (Luciano et al., 2018), generating a 5′-p terminus from the 5′-pp product of ApaH action, thus allowing further rapid 5′-degradation by the RNase E endonuclease (Farr et al., 1989; Deana et al., 2008; Monds et al., 2010; Luciano et al., 2019). The 5′ cap can also influence the rate of decapping, with RppH and ApaH showing distinct activities on methylated caps. ApaH can remove all Ap_4_N caps, while RppH cannot cleave methylated caps (Figure 2; Hudeček et al., 2020).

In unstressed *E. coli* cells, deletion of *ApaH* (but not *RppH*) also promotes increased Np_n_ capping (Luciano et al., 2019). Thus, differences in the relative concentrations of Ap_4_Ns, the promoter sequence-dependent use of different caps and differences in the efficiencies of Ap_n_N cap removal all combine to suggest that regulation of ApaH activity, whether by thiol inactivation or by other means, could provide a powerful means of controlling mRNA stability and differential gene expression. Np_n_ capping may explain the phenotypic changes previously associated with increased intracellular Ap_4_N levels in bacteria including reduced motility, altered biofilm formation, reduced ability to invade mammalian cells, and uncoupling of DNA replication and cell division (Farr et al., 1989; Nishimura et al., 1997; Ismail et al., 2003; Deana et al., 2008; Monds et al., 2010). Each phenotype is associated with *ApaH* deletion or disruption although loss of cellular invasion is also commonly seen in bacteria lacking the nudix hydrolase RppH/NudH/YgdP/IalA. This suggests that precise regulation of intracellular Ap_4_N levels is required to prevent aberrant signaling. Such control is revealed in stressed cells where ApaH hydrolase acts as both sensor and effector through regulation of 5′ methyl-Np_4_ capped mRNA stability.

### Ap_4_A-Mediated Regulation of Gene Expression in Eukaryotes

**FIGURE 3:**
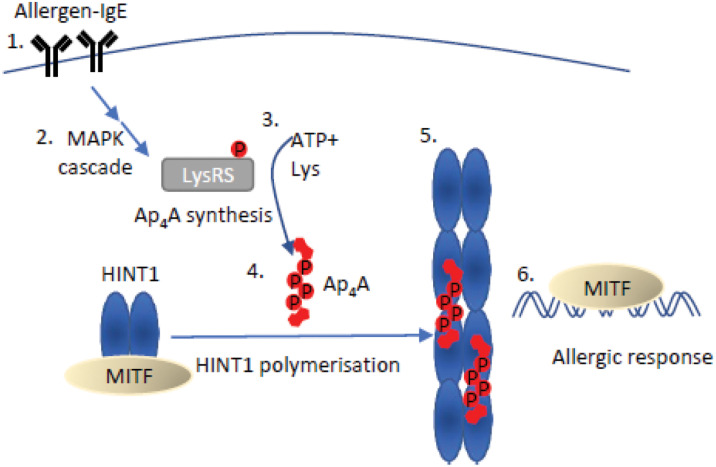
Mechanism of Ap_4_A mediated regulation of HINT1-MITF. An allergen bound to IgE **(1)** stimulates the mitogen-activated protein kinase (MAPK) cascade **(2)**, resulting in the phosphorylation of LysRS on serine 207. Phosphorylation of LysRS increases Ap_4_A synthesis 3-fold **(3)** (Yannay-Cohen et al., 2009). The increase in Ap_4_A concentration causes dissociation of the MITF-HINT1 complex as Ap_4_A and MITF compete for the same binding site on HINT1. Ap_4_A binding to HINT1 promotes its polymerization **(5)**. Ap_4_A bridges the HINT1 dimer–dimer interface promoting association with the adjacent dimer facilitating their polymerization (Yu et al., 2019). Following HINT1 polymer formation, MITF translocates to the nucleus to promote transcription of target genes and initiate an allergic response **(6)** (Yu et al., 2019).

An early indication of a direct signaling role for Ap_4_A in eukaryotic cells was the regulation of MITF-mediated transcription in mast cells in response to allergen activation. HINT1 is an Ap_4_A-binding member of the HIT protein family. HINT1 interacts with microphthalmia-associated transcription factor (MITF) in the absence of any stimulus and represses its activity. When mast cells are activated by IgE-allergen binding to its cognate receptor, this enhances ERK1/2 activity promoting phosphorylation of LysRS on S207 (Yannay-Cohen et al., 2009). LysRS is an aminoacyl tRNA synthetase capable of efficient Ap_4_A synthesis (Zamecnik et al., 1966). Importantly, LysRS binds MITF to form a multiprotein complex with HINT1 which causes Ap_4_A to be produced in close proximity to HINT1, facilitating Ap_4_A-HINT1 binding (Lee et al., 2004). Activation of a mitogen activated protein kinase (MAPK) signaling cascade promotes phosphorylation of LysRS (pS-207) resulting in its translocation to the nucleus. LysRS aminoacylation activity is inhibited by phosphorylation of S207 that concomitantly increases its Ap_4_A synthetic activity 3-fold. The increase in Ap_4_A levels promotes oligomerization of HINT and release of MITF (Figure 3; Razin et al., 1999; Lee et al., 2004; Yannay-Cohen et al., 2009).

The dissociation of the HINT1-MITF complex can also be induced in melanoma cells through post-translational modification of HINT1 residues surrounding the MITF-HINT1 interface, which may allow prolongation of MITF-controlled processes (Motzik et al., 2017). Importantly, these residues are proximal to the Ap_4_A binding site in HINT1, suggesting a competitive mechanism between Ap_4_A and MITF for HINT1 binding (Figure 3; Yu et al., 2019). The specificity of this transcriptional response for a dinucleoside tetraphosphate arises from the specific requirements for oligophosphate chain length for the oligomerization of HINT1. Despite Ap_3_A showing reduced inhibition of MITF activity (though less than Ap_4_A), neither Ap_3_A nor Ap_5_A could initiate HINT1 polymerization (Yu et al., 2019). The HINT1-Ap_4_A crystal structure revealed the structural basis for the specific chain length for optimal Ap_4_A-HINT interactions, as Ap_3_A chain length was insufficient to interact at nucleotide binding pockets and Ap_5_A binding required a contortion of the phosphodiester linkages (Yu et al., 2019). Recently some doubt has been expressed regarding the ability of HINT1 to bind Ap_4_A, but this could reflect differences in analytical techniques and a lack of post-translational modifications in the HINT1 used (Strom et al., 2020).

Microphthalmia-associated transcription factor is involved in signaling in a wide variety of processes including DNA repair and cell survival, as well as cell proliferation and invasion (Goding and Arnheiter, 2019). After Ap_4_A-mediated dissociation from HINT1, MITF is free to bind E box enhancer elements in the promoter region of specific genes, resulting in stimulation of their transcription (Lee et al., 2004). To prevent deregulation of MITF-target genes, levels of Ap_4_A are subsequently down-regulated by hydrolysis, demonstrated by the changes in expression of rat MITF- (and USF2-) target genes when Nudt2, the major Ap_4_A hydrolase, is knocked down (Carmi-Levy et al., 2008). Similarly, MITF localization to the nucleus and increased transcription of MITF-target gene TRACP5 has also been demonstrated in murine dendritic cells that have a floxed *Nudt2* gene and therefore a 30-fold increase in Ap_4_A levels (La Shu et al., 2019). Further evidence for the importance of precise control of Ap_4_A levels was demonstrated in bone marrow-derived dendritic cells (BMDCs) with floxed *NUDT2*. This leads to greater motility and increased proliferation of CD8+ T cells that require cross-antigen presentation, however, this only correlates with increased MITF target gene expression and is not necessarily directly caused by it (La Shu et al., 2019). The structural, biochemical and cellular analysis of the role of Ap_4_A in the regulation of HINT1-MITF demonstrate that Ap_4_A does indeed perform a *bona fide* signaling role that can precisely regulate transcriptional programs in specific cellular lineages.

### Ap_4_A Mediated Regulation of the cGAS-STING Pathway

**FIGURE 4:**
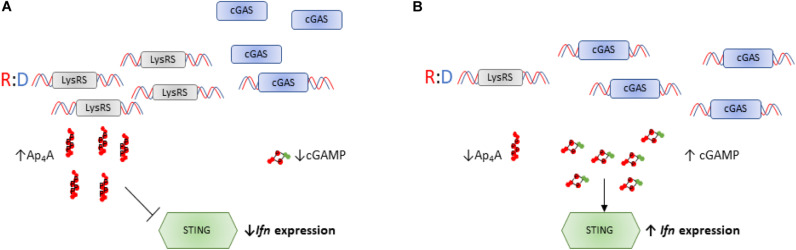
Ap_4_A regulates activity of the cGAS-STING pathway. **(A)** LysRS suppresses activation of cGAS through two distinct mechanisms: (i) LysRS competes with cGAS for binding DNA hybrids, reducing cGAS:hybrid interactions and therefore reducing synthesis of cGAMP (ii) LysRS synthesizes Ap_4_A which binds to STING in the cGAMP binding pocket, delaying interactions between cGAMP and STING (Guerra et al., 2020). This therefore inhibits STING activation and reduces interferon expression. **(B)** When LysRS levels are low, it cannot successfully compete with cGAS for R:D hybrid binding. This results in efficient cGAS/R:D hybrid interactions and increased cGAMP production (Guerra et al., 2020). High intracellular cGAMP and low intracellular Ap_4_A concentrations results in efficient activation of STING and increased interferon expression.

A second transcriptional program influenced by LysRS-mediated Ap_4_A accumulation is the STING-dependent inflammatory response. The cyclic GMP-AMP (cGAMP) synthase (cGAS)-stimulator of interferon (IFN) genes (STING) pathway is a component of the innate immune response that contributes to recognition of viral nucleic acid within cells (Almine et al., 2017). In response to viral nucleic acid, cGAS generates cGAMP that activates STING and downstream transcription factors such as STAT6 and IRF3 via TANK-binding kinase 1 leading to expression of type 1 interferons. Evidence has been presented that LysRS plays a role in suppressing cGAS activation via two complementary mechanisms, one of which involves the synthesis of Ap_4_A (Guerra et al., 2020). First, LysRS competes with cGAS for binding to RNA-DNA hybrids thus reducing the synthesis of cGAMP, and secondly LysRS synthesizes Ap_4_A which binds to the cGAMP binding pocket of STING preventing its activation (Figure 4; Guerra et al., 2020).

The modeling of cGAS-Ap_4_A interactions revealed contortions of the phosphate linker suggesting that chain length may affect function, although no analysis was performed using other Np_n_Ns. There was also an effect of extracellular Ap_4_A and a non-hydrolyzable analog in reducing IFN*β* mRNA production. Interestingly, in the context of innate host defense to viral nucleic acids, Ap_n_A, and in particular Ap_5_A, are sub-micromolar inhibitors of the pancreatic ribonuclease superfamily including eosinophil-derived neurotoxin, an important antiviral protein (Kumar et al., 2003; Baker et al., 2006). Also, the NUDT2 Ap_4_A hydrolase has been reported to interact with the SARS coronavirus 7a protein (Vasilenko et al., 2010). The effect of this interaction is unknown but if it led to NUDT2 inhibition and a rise in Ap_4_A, this could provide a means for the virus to prevent STING activation and add to the repertoire of mechanisms used by coronaviruses to evade the innate host immune response (Maringer and Fernandez-Sesma, 2014).

The regulation of cGAS-STING by Ap_4_A and the observed reduction in IFN signaling by high Ap_4_A levels is entirely consistent with the effect of *NUDT2* disruption on the transcriptome of KBM-7 CML cells (Marriott et al., 2016). Inactivation of the NUDT2 Ap_4_A hydrolase led to a 175-fold increase in intracellular Ap_4_A and major changes in the transcriptome involving over 6,000 differentially expressed genes. Among the down-regulated gene sets were those associated with interferon responses, pattern recognition receptors and inflammation while functions associated with MHC class II antigens featured among the up-regulated genes. For example, in agreement with the study above, IFN*β* was down-regulated 15-fold. Tryptophan catabolism was also strongly down-regulated as were genes involved in tumor promotion, particularly in the epithelial-mesenchymal transition, proliferation, invasion and metastasis. No single cause was identified for these changes but possibilities discussed included LysRS-mediated HINT1 activation, inhibition of protein kinases and other enzymes/proteins, autocrine activation of purinoceptors and chromatin remodeling. However, given the recently discovered role of dinucleotide caps in regulating mRNA stability in prokaryotes, a similar alternative function for increased Ap_4_N in regulating specific mRNA turnover in mammalian cells cannot be ruled out. Four different classes of enzyme possess decapping ability in eukaryotes (Kramer and McLennan, 2019). The “classical” decapping enzyme is DCP2 (NUDT20), a nudix family hydrolase. However, both NUDT3 and NUDT16 appear to decap specific subsets of mRNAs while several other nudix hydrolases, including NUDT2, can hydrolyze a variety of canonical and non-canonical caps *in vitro* and also bind RNA (Grudzien-Nogalska and Kiledjian, 2017; Ray and Frick, 2020; Sharma et al., 2020). DXO enzymes can remove caps from incompletely capped mRNAs as part of the cap quality control pathway and also NAD, FAD and dephospho-CoA caps (Doamekpor et al., 2020), while HIT family enzymes degrade capped RNA fragments that are remnants of the 3′ to 5′ decay pathway. Finally, the major enzyme involved in decapping of mRNA transcripts in *Trypanosoma brucei*, ALPH1, is an ApaH-like phosphatase that cleaves the unusual caps found in this organism (Perry et al., 1987; Kramer, 2017). Thus, there are several candidates that could perform Np_4_-cap removal in eukaryotes, if Ap_4_N are capable of initiating synthesis of mRNA. This modification has not yet been identified in eukaryotes, but this remains an intriguing possibility for the regulation of mRNA stability in stressed cells. Such a mechanism could go a long way to explain the dramatic changes observed in the transcriptome of NUDT2 knockout cells (Marriott et al., 2016).

There has also been a suggestion that Ap_4_A could regulate the 3′ end processing of pre-mRNA. Cleavage factor I_m_ (CF I_m_) defines the site of 3′ poly(A) addition by inducing both poly(A) addition and cleavage (Brown and Gilmartin, 2003). The 25 kDa subunit of CF I_m_ (CF I_m_25, F5, NUDT21) is a catalytically inactive nudix protein that binds Ap_4_A as a homodimer in a manner that excludes RNA suggesting that Ap_4_A binding may somehow control CF I_m_ activity (Yang et al., 2010). CF Im is regulated by ATP binding that both inhibits and activates 3′ cleavage of pre-mRNAs in a concentration dependent manner. Ap_4_A has a higher affinity for CF Im relative to ATP (Coseno et al., 2008). However, unlike ATP, Ap_4_A does not affect ATP-stimulated cleavage (Khleborodova et al., 2016). Thus, the evidence that Ap_4_A contributes to regulation of the 3′ end of mRNA remains questionable.

### Ap_4_A as a DNA Damage-Associated Regulator of Eukaryotic DNA Replication

The synthesis of Ap_4_A in response to genotoxic stress suggests possible roles in the DNA damage response, DNA replication and cell cycle progression. However, there are conflicting reports regarding the role of Ap_4_A in the regulation of DNA replication. Initially, Ap_4_A was found to initiate DNA replication when added to permeabilized G1-arrested BHK cells and was later reported to increase up to 1000-fold prior to S-phase entry (Grummt, 1978, 1979; Weinmann-Dorsch et al., 1984; Zourgui et al., 1984). It also stimulated DNA replication in microinjected *Xenopus laevis* oocytes (Zourgui et al., 1984). However, other reports failed to confirm these findings (Garrison et al., 1986; Moris et al., 1987; Orfanoudakis et al., 1987; Perret et al., 1990). In support of a role in initiation, Ap_4_A was found to associate with a DNA polymerase α complex that synthesizes the RNA primers required for DNA replication (Grummt et al., 1979; Rapaport et al., 1981) and an Ap_4_A-binding protein was identified in this complex in calf thymus and HeLa cells (Grummt et al., 1979; Rapaport et al., 1981). This protein was later resolved into 45 kDa (A1) and 22 kDa (A2) polypeptides, with Ap_4_A binding to the larger polypeptide (Baxi et al., 1994) but it still remains unidentified. In the context of DNA repair, another nuclear Ap_4_A-binding protein is uracil DNA-glycosylase/glyceraldehyde-3-phosphate dehydrogenase (UDG/GAPDH), which interacts with several DNA repair factors (Baxi and Vishwanatha, 1995; Azam et al., 2008; Kosova et al., 2017). Ap_4_A was also able to act as a preformed primer for DNA polymerase α *in vitro* (Zamecnik et al., 1982). This draws an interesting parallel with the use of Ap_n_N as primers for RNA polymerase in the production of 5′ Np_4_ mRNA caps in *E. coli* as discussed earlier. The use of non-canonical initiating nucleotides is shared by the *E. coli* DnaG primase, which can initiate primer synthesis with NADH and FAD (Julius et al., 2020). The use of novel nucleotides for the initiation of RNA primer synthesis has important implications for the potential role of Ap_4_A and other Ap_4_N in the regulation of the initiation phase of DNA replication. However, the reconstitution of origin firing *in vitro* using 16 recombinant proteins (Yeeles et al., 2015; Aria and Yeeles, 2019) provides no opportunity for the addition or production of Ap_4_A. Hence, at least for the minimal DNA replication machinery from *S. cerevisiae*, Ap_4_A is not essential for the initiation phase of DNA replication; however, this does not exclude a function as an initiator in some specific contexts.

**FIGURE 5:**
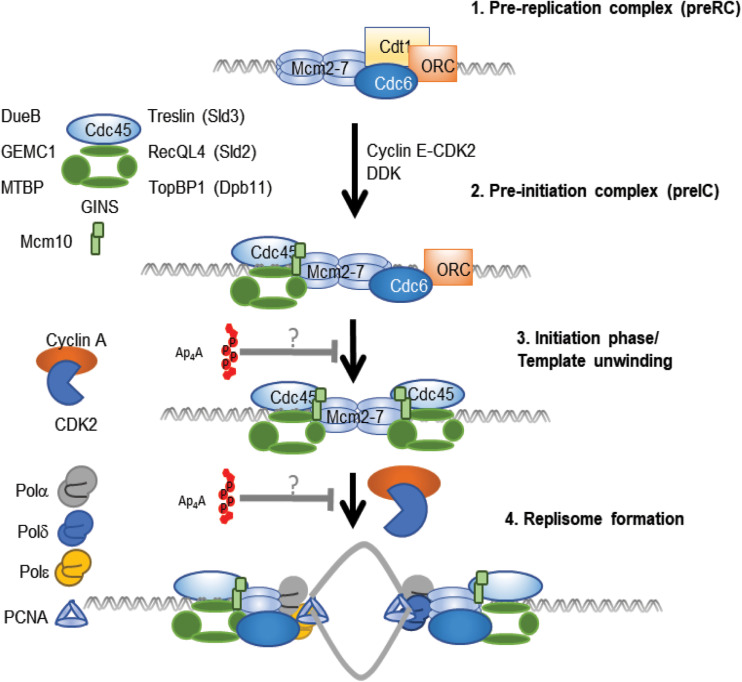
Ap_4_A-mediated inhibition of DNA replication model. The first step of DNA replication involves the formation of the Pre-replication complex **(1)**, where Cdc6 and the origin recognition complex (ORC) promote formation of the Mcm2-7 double hexamer through the recruitment of Cdt1 molecules (Takara and Bell, 2011). This is followed by activation of the complex through further recruitment of several proteins to form the Pre-initiation complex (pre-IC) **(2)** (Pacek et al., 2006). With Ap_4_A at low levels, Cyclin A and Cdk2 would initiate DNA replication and template unwinding **(3)**, resulting in the recruitment of several polymerases and PCNA to form the replisome **(4)** (Yao and O’Donnell, 2010). Ap_4_A inhibits the initiation phase of DNA replication (Marriott et al., 2015). There is potential for Ap_4_A to inhibit either the 3rd or 4th step in this process. However, the precise mechanism and the protein(s) that Ap_4_A associates with to inhibit DNA replication remains to be determined. This is highlighted with “?” in figure to highlight that Ap4A may act to inhibit the pre-IC to template unwinding step or prevent activation of the replisome.

In contrast to results supporting the promotion of DNA replication by Ap_4_A, recent evidence suggests that Ap_4_A inhibits the initiation phase of DNA replication. An early indication of an inhibitory effect of Ap_4_A during initiation was the abrupt fall in Ap_4_A before the onset of each S-phase in sea urchin embryos (Morioka and Shimada, 1985). More recently, Ap_4_A was found to directly inhibit the initiation phase, but not the elongation phase of DNA replication in a cell-free replication system comprising mouse cell nuclei and cytoplasmic factors. Initiation was inhibited maximally by about 70–80% in nuclei treated with >20 μM Ap_4_A, a level consistent with that found after treatment with sub-lethal doses of cross-linking agents (Marriott et al., 2015). Ap_3_A, Ap_5_A, Gp_4_G, and ADP-ribosylated Ap_4_A were without significant effect, demonstrating a high degree of specificity in this response. As Ap_4_A concentration increases under a number of different stresses, this suggests that Ap_4_A may be involved in inhibiting DNA replication initiation when the cell is under stress in order to preserve genome integrity during recovery (Figure 5).

Of the dinucleotides tested only Ap_4_A has so far been found to inhibit DNA replication. The effect of Ap_4_A in the HINT1-MITF (Yu et al., 2019) and cGAS-STING (Guerra et al., 2020) pathways involves competitive inhibition, where Ap_4_A competes for ATP binding pockets. Such a mechanism offers several opportunities for the regulation of DNA replication where ATP is required for origin specification, kinase activity, clamp loading, helicase activity, primer synthesis and elongation of the DNA template. Thus, Ap_4_A could act by inhibition of an ATP-dependent step during the initiation phase of DNA replication. The model presented above highlights that there are only two phases that could be affected by Ap_4_A in *in vitro* cell free DNA replication assays that utilize replication licensed nuclei. The CDK dependent initiation of DNA replication involves recruitment and activation of the replicative helicase and replisome formation indicating several factors that could be affected during this process (Figure 5). Identification and analysis of the proteins with which Ap_4_A associates could reveal relevant targets for Ap_4_A-mediated competitive inhibition.

The observed role for Ap_4_A in the initiation phase of DNA replication may also be supported by the finding that direct exposure of T47D and MCF7 breast cancer cells to 100 μM Ap_4_A slows their proliferation rate while increased NUDT2 expression (with a resulting reduction in Ap_4_A) increases their proliferation (Oka et al., 2011). However, other studies suggest that cells can tolerate greatly increased intracellular Ap_4_A without a noticeable effect on proliferation (Murphy and McLennan, 2004; Marriott et al., 2016). Thus, the cell-free system that identified the inhibition of replication initiation by Ap_4_A may reflect a response to acute changes to Ap_4_A levels that is overcome under chronic stress conditions such as in the NUDT2 knockout KBM7 cell lines.

Interestingly, various dNp_n_dN can act as elongation substrates for some DNA polymerases, including *E. coli* Pol I, human pol α and β, and HIV reverse transcriptase with the elimination of an NTP rather than the usual PPi (Victorova et al., 1999). The reverse reaction analogous to pyrophosphorolysis can remove 3′ chain terminating dideoxynucleotides such as those incorporated from anti-HIV drugs to generate dinucleoside polyphosphates (Table 1; Smith et al., 2005; Dharmasena et al., 2007). How these findings relate to the inhibition of DNA replication initiation by Ap_4_A is currently unclear but they do illustrate that Ap_n_N and other Np_n_N are not excluded from the replication apparatus and may be generated as signals and/or primers by DNA polymerases at stalled replication forks (Figure 5) Therefore, a more detailed understanding of the potential roles of other Ap_4_N molecules in the DNA replication process *in vivo* is required.

### Additional Ap_4_A Binding Partners and Downstream Signaling

The association of Ap_4_A with HINT1 and STING and the subsequent downstream events would appear to satisfy sufficient criteria to classify Ap_4_A as a *bona fide* signaling molecule in eukaryotes. Is there any evidence for other binding proteins and pathways? Despite much interest and research, Ap_4_A binding partners with clear roles in signaling pathways remained elusive for many years. Initially, several stress proteins in *E. coli* were found to interact with Ap_4_A, including DnaK, GroEL, ClpB, C45 and C40 (Johnstone and Farr, 1991; Fuge and Farr, 1993). Binding of Ap_4_A to the GroEL chaperone was later found to involve sites distinct from the known ATP/ADP binding sites and a novel role in promoting substrate release from the substrate-GroEL complex was suggested (Tanner et al., 2006). The hsp70 family are homologs of the DnaK protein and several Ap_4_A-binding hsp70 proteins have also been found in murine brain lysates by magnetic biopanning using biotin-conjugated Ap_4_A, which demonstrates that Ap_4_A-stress protein binding occurs across different kingdoms (Guo et al., 2011). However, it is unclear whether Ap_4_A is acting as anything other than an ATP analog in binding to these proteins (Despotović et al., 2017). Several other Ap_4_A-binding proteins have been consistently found using these methods (Guo et al., 2011; Azhar et al., 2014) but with no obvious clue as to function of the interaction. One of these is inosine-5′-monophosphate dehydrogenase (IMPDH), which contains a cystathionine-*β*-synthase (CBS) domain. These domains are often found in pairs forming a cleft to which nucleotides can bind, known as the Bateman domain (Baykov et al., 2011). Several enzymes contain CBS domains including CBS, IMPDH and AMP-activated protein kinase (AMPK) (Anashkin et al., 2017). Of these, Ap_4_A was found to bind bacterial IMPDH, an important control enzyme in the biosynthesis of guanine nucleotides with three allosteric nucleotide binding sites, and a potential role for Ap_4_A in regulating cell proliferation via IMPDH was suggested (Guo et al., 2011; Anashkin et al., 2017). However, the binding affinity of Ap_4_A is not much greater than that of ATP, probably because it can only bind to a single site, and its physiological role has been questioned (Despotović et al., 2017; Fernández-Justel et al., 2019). On the other hand, because of their increased polyphosphate chain length, Ap_5_A and Ap_6_A can span canonical sites 1 and 2 in *Ashbya gossypii* IMPDH (*Ag*IMPDH) and submicromolar levels of these nucleotides can reverse inhibition of *Ag*IMPDH induced by millimolar GDP. Due to a slight structural difference, a similar effect was only found for Ap_5_A with human IMPDH (Fernández-Justel et al., 2019). In contrast, Ap_5_G potentiates GTP/GDP-mediated allosteric inhibition of both IMPDH enzymes by binding to sites 1 and 2 with the G in site 2, which enhances GDP/GTP binding to site 3. These effects suggest a role for Ap_5_N in IMPDH regulation.

The suggested lack of relevance of Ap_4_A as a protein ligand highlights a particular problem with this nucleotide in that it may often simply bind to proteins via a single adenosine moiety as an ATP analog. Binding specificity or at least a favorable comparison of binding constants is necessary before biological relevance can be proposed. For example, some family II pyrophosphatases, such as a pyrophosphatase from *Clostridium perfringens*, contain two CBS domains (CBS-PPases) unusually linked by a DRTGG domain (Tuominen et al., 2010). These pyrophosphatases are inhibited by AMP binding to each CBS monomer and can be activated through Ap_4_A binding to Met114 and Tyr278 of CBS domains 1 and 2, respectively, residues that are present in pairs at the subunit interface (Tuominen et al., 2010). Ap_n_A, where *n* = 4–6, can bind the CBS domains of pyrophosphatases from *C. perfringens, Desulfitobacterium hafniense* and *Clostridium novyi* non-cooperatively with nanomolar affinities, increasing their activity between 5- and 30-fold dependent on species (Anashkin et al., 2015). The DRTGG domain is likely to be important in this interaction, as CBS-PPase activities in *Eggerthella lenta* and *Moorella thermoacetica*, which do not possess a DRTGG domain, were not affected by the diadenosine polyphosphates (Anashkin et al., 2015). This high affinity binding suggests that Ap_n_As can control CBS-PPase activity and hence regulate pyrophosphate levels in bacteria.

Other intracellular signaling pathways can be affected by changes in Ap_n_A levels. When pancreatic islets were incubated with glucose to induce insulin release, the intracellular concentrations of Ap_4_A and Ap_3_A were found to rise 70- and 30-fold to 14 and 11 μM, respectively. Such concentrations are sufficient to close K_ATP_ channels in *β*-cell membrane patches resulting in membrane depolarization and insulin release, suggesting that these dinucleotides act as second messengers to mediate glucose-induced blockade of *β*-cell K_ATP_ channels (Ripoll et al., 1996; Martín et al., 1998). This may occur through Ap_n_A-mediated inhibition of adenylate kinase, a determinant of K_ATP_ channel activity (Stanojevic et al., 2008). A similar role for Ap_5_A in regulating K_ATP_ channel activity in cardiac myocytes has also been proposed (Jovanovic et al., 1998). Micromolar levels of Ap_n_A are also able to activate ryanodine receptors in brain and heart endoplasmic reticulum and have been proposed as physiological regulators of these calcium release channels (Shepel et al., 2003; Song et al., 2009).

### Ap_n_N Signaling Through Internalization of Extracellular Pools

In addition to the intracellular signaling mechanisms discussed here Ap_n_N are involved in a number of extracellular signaling networks (Jankowski et al., 2009). These nucleotides are mostly released from stores such as adrenal chromaffin granules and platelet dense granules into the extracellular space where generally they operate via cell-surface purinergic receptors. Endothelial cells may also release Ap_n_N, and in particular Ap_4_U, synthesized by an unusual activity of the cytoplasmic domain of vascular endothelial growth factor receptor 2 (Jankowski et al., 2013). However, some extracellular Ap_n_N may become internalized and operate intracellularly. The first indication of this was the internalization of radiolabeled Ap_4_A by bovine aortic endothelial cells via a two-step mechanism (Hilderman and Fairbank, 1999). Some studies that show an intracellular response to added Ap_4_A, e.g., STING inhibition (Guerra et al., 2020), must presumably rely on such a mechanism but it remains unidentified. Similarly, internalization may be behind changes in gene expression induced by external Np_n_N in plants. When Ap_4_A was added to *Arabidopsis thaliana* seedlings there was an increase in the expression of several genes and activity of proteins involved in early steps of the phenylpropanoid pathway (Pietrowska-Borek et al., 2011). The phenylpropanoid pathway is triggered in response to stress and results in the production of stilbenes such as resveratrol, as well as flavonols and lignins (Dixon and Paiva, 1995). Importantly, 4-Coumarate:CoA ligase, known to synthesize Ap_4_A, is one of the enzymes in the phenylpropanoid pathway whose activity was increased by the addition of Ap_4_A (Pietrowska-Borek et al., 2003, 2011). More recently it was also shown that a variety of other Np_n_Ns can modulate the phenylpropanoid pathway in *Vitis vinifera* cv. Monastrell suspension cultured cells, meaning that induction of the response is not specific to Ap_4_A (Pietrowska-Borek et al., 2020b). Interestingly, certain variations in nucleotide have opposing effects. Ap_3_A induced accumulation of the stilbene *trans-*resveratrol, a response that was also induced by Up_3_U, Ap_3_U, Up_4_U as well as others; however, Cp_3_C, Cp_4_C, Ap_3_C, and Ap_4_C all inhibited stilbene synthesis, causing stilbene levels to be 300–400-fold lower than controls (Pietrowska-Borek et al., 2020b). Despite this evidence suggesting a role for Np_n_Ns in regulation of the phenylpropanoid pathway, no receptors or signaling pathways have been identified. It has been suggested that pathogens may synthesize the Np_n_Ns, which could be transported into the cell by some unknown mechanism involving plasma membrane transporters (Pietrowska-Borek et al., 2020a).

### Roles for Dinucleoside Polyphosphates Other Than Ap_4_A

While most attention over the years has focused on Ap_4_A, it is clearly not the only Np_n_N with biological activity. Aminoacyl-tRNA synthetases can adenylate a wide variety of nucleotide acceptors both *in vitro* and *in vivo* with varying efficiencies to yield products such as Ap_3_A, Ap_3_G, Ap_4_C, Ap_5_U, and Ap_3_Gp_2_ (from ppGpp). Non-nucleotide acceptors such as tri- and tetrapolyphosphate, thiamine pyrophosphate and isoprenyl polyphosphates can also be adenylated, the former pair yielding p_4_A and p_5_A which can be further adenylated to give Ap_5_A and Ap_6_A (Plateau and Blanquet, 1982; Brevet et al., 1989; McLennan, 1992; Sillero et al., 2009). Ap_5_A is a potent “multisubstrate” inhibitor of adenylate kinases (Lienhard and Secemski, 1973) and is frequently used *in vitro* to inhibit this enzyme although whether it can do so *in vivo* has not been properly investigated. Non-adenylated Np_n_N such as Cp_4_C, Gp_4_G, Cp_4_U etc., have also been found in *E. coli* and *S. cerevisiae* and increase in concentration after oxidative and thermal stress (Coste et al., 1987). These could be generated by a number of enzymes including Ap_4_A phosphorylases, UTP-glucose-1-phosphate uridylyltransferase and capping guanylyltransferases and could also conceivably arise non-enzymically (Wang and Shatkin, 1984; McLennan, 2000; Guranowski et al., 2004; Table 1). Many of the more unusual derivatives probably are damage metabolites. However, functions have been assigned to some of the homodinucleotides. The potential for Ap_5_A to regulate IMPDH activity and the ability of Ap_3_A, Up_3_U, Up_4_U, and Cp_4_C to differentially regulate stilbene synthesis in *V. vinifera* have already been described (Pietrowska-Borek et al., 2020b). In mammals, Ap_3_A is a ligand of the FHIT tumor suppressor protein. It has hydrolase activity and this controls the intracellular level of Ap_3_A (Barnes et al., 1996; Murphy et al., 2000). Ap_3_A binding but not hydrolysis is required for its tumor suppressive function (Pace et al., 1998). FHIT has been implicated in several pro-apoptotic pathways but the precise role of Ap_3_A binding remain elusive (Okumura et al., 2009). Gp_4_G and Gp_3_G have unique roles as a source of purines and energy in encysted embryos of the brine shrimp *Artemia franciscana* and closely related crustacea and are synthesized by a unique Gp_4_G synthetase (Liu and McLennan, 1994).

What about the various heterodinucleotides and molecules containing a non-nucleotide moiety? Do they have specific roles beyond those recently revealed in mRNA capping? Some of the common Ap_4_A assays, such as those based on bioluminescence, will also record and include other adenylated dinucleotides in the “Ap_4_A pool” and they are often ignored as being “minor” or of lesser importance as they are usually present at lower concentrations. Where they have been measured separately they tend to behave in a manner similar to Ap_n_A, e.g., the lack of cell cycle fluctuation and increase in response to dinitrophenol treatment displayed by both Ap_4_A and Ap_4_G in *Physarum polycephalum* (Garrison et al., 1986). Nevertheless, as regulation or deletion of hydrolases such as NUDT2, FHIT, ApaH and RppH will alter their levels alongside Ap_4_A, Ap_3_A etc., possible differential or even unique activities should not be discounted. The spanning of two nucleotide-binding sites in IMPDH by Ap_5_G (Fernández-Justel et al., 2019) is a good example of the potential regulation of enzyme activities by Ap_n_N. Ap_4_dT and Ap_5_dT can inhibit thymidine kinase, the higher potency of the latter showing the importance of polyphosphate chain length (Bone et al., 1986). Additionally, the uridine kinase of Ehrlich ascites tumor cells is inhibited by Ap_4_U and various deoxynucleotide kinases of *Lactobacillus acidophilus* by micromolar concentrations of Ap_4_dC, Ap_4_dT and Ap_4_dG as appropriate (Cheng et al., 1986; Ikeda et al., 1986). An adenylated derivative of isopentenyl pyrophosphate, unfortunately called ApppI (Ap_3_I) where I is not inosine, accumulates in cells where the mevalonate pathway has been inhibited by nitrogen-containing bisphosphonates and inhibits the mitochondrial adenine nucleotide translocase resulting in apoptosis, providing a mechanism for the action of these drugs (Mönkkönen et al., 2006). Thiaminylated ATP (AThTP, Ap_3_Th) accumulates in *E. coli* specifically in response to carbon starvation and may signal starvation stress. It has also been detected in plant and animal tissues and can inhibit the enzyme PARP-1 (Bettendorff et al., 2007; Tanaka et al., 2011). The potency of enzyme inhibition caused by many of these dinucleotides does show the problems they could cause as unregulated damage metabolites but the harnessing of this ability for more beneficial purposes has yet to be ruled out. Bacterial cGAS-like enzymes have recently been found to synthesize a wide range of purine- and pyrimidine-containing cyclic di- and trinucleotides with apparent signaling ability (Whiteley et al., 2019) and perhaps the same could yet be true for many Np_n_N species.

## Discussion and Future Perspectives

Compared to other signaling molecules such as cyclic dinucleotides, “magic spot” guanine nucleotides and inositol polyphosphates, dinucleoside polyphosphates have consistently confounded attempts to define their biological roles, leading to the suggestion that they are functionless damage metabolites. However, recent evidence from studies in both prokaryotes and eukaryotes has finally established intracellular roles for the adenosine-containing members of this family, generally as part of the cellular response to molecular stress. The number of enzymes potentially able to regulate these Ap_4_N through synthesis and degradation has also been expanded.

Whether one can apply the labels “signal” or “messenger” to the stress-promoted use of Ap_4_N as RNA polymerase initiators in prokaryotes depends on how far one should be bound by the commonly used qualifying definitions of these terms, but they can at least be viewed as active participants in the regulation of mRNA stability under conditions of oxidative stress. Moreover, as these conditions lead directly to a rise in Ap_4_N levels via ApaH inactivation, the epithet “signal” seems reasonable in this context. Active participation of Ap_4_N as primers for DNA replication leading either to activation, inhibition or rescue of replication depending on other factors or experimental conditions may be an analogous mechanism and requires further investigation. In addition to any participatory roles in mammalian cells, however, the activation of MITF-mediated transcription and modulation of STING-mediated interferon responses by binding of Ap_4_A to a specific target protein clearly satisfy sufficient of the standard signaling criteria to allow the inclusion of diadenosine polyphosphates within the superfamily of highly phosphorylated small signaling molecules.

Thus, the functions of dinucleoside polyphosphates may be classified into two distinct modes of action: first, as primers for nucleic acid synthesis, and secondly as competitive ligands of ATP-binding sites on target proteins. The distinct modes of action for different members of the Ap_n_N family may introduce considerable flexibility into the responses. For example, the use of various Ap_n_N species for initiation at different bacterial promoters can affect the stability of specific mRNAs depending on the relative concentrations of available Ap_n_N and their rates of removal from the 5′ ends. The regulation of MITF activity may be exclusive to Ap_4_A as the mechanism requires binding to two adjacent ATP-specific binding pockets in HINT1 dimers. On the other hand, the competition between cGAMP and Ap_4_A for binding to STING involves a nucleotide binding pocket that can accommodate both guanosine and adenosine, raising the question as to whether other Ap_4_N and in particular Ap_4_G could also mediate this function. Thus, there is now a clear need to discriminate between the activities of Ap_4_A and other Ap_n_N and even Np_n_N *in vivo*. Several of the recently described pathways, such as mRNA capping and the regulation of gene expression by the internalization of plant dinucleotides, have highlighted the possibility that other Ap_n_N and Np_n_N may have roles in the signaling of stress across different species. By analogy, there is potential for Ap_n_N to perform a stress-related non-canonical mRNA cap function in eukaryotes, but this has not yet been demonstrated.

The importance of Ap_3_A to the tumor suppressor function of the FHIT protein is already well established, although its precise role is still unclear. The possible relevance *in vivo* of other Ap_3_N and Np_3_N, all substrates for the FHIT hydrolase activity, is also unknown. Despite their observed functions in prokaryotic transcriptional control, individual Ap_n_N have yet to be reliably detected and quantified in mammalian cells. This will require improved detection technologies. The use of coupled enzyme assays (typically luciferase-based) to determine “Ap_4_A” levels is suboptimal as they do not discriminate between Ap_4_A and other Ap_4_N as ATP can be produced from either by the coupled Ap_4_A hydrolase. This has potentially limited our understanding and appreciation of individual Ap_4_N in mammalian cells. Advances in detection via liquid chromatography/tandem mass spectroscopy have aided the quantitation of different Ap_n_N and have led to the identification of Ap_4_U in extracellular environments (Schulz et al., 2014; Götz et al., 2019; Ji et al., 2019; Fung et al., 2020). Establishing new LC-MS/MS methods for the analysis of individual intracellular Ap_n_N and other Np_n_N will clarify the biological relevance of these dinucleotides in stress responses in mammalian systems.

Furthermore, it has not yet been determined which enzyme(s) in addition to LysRS may be responsible for the synthesis of individual Ap_n_N under different circumstances *in vivo.* The production of Ap_n_N *in vitro* usually involves either an acyl-adenylate or enzyme-adenylate intermediate, with the adenylate being subsequently transferred to an acceptor nucleotide. All of the enzymes previously described can do this *in vitro* to some extent and are therefore candidates. Moreover, the cytoplasmic domain of VEGFR2 appears able to generate various dinucleotides by direct condensation of two mononucleotides without an intermediate and could be a paradigm for the intracellular production of Ap_n_N and Np_n_N by other receptor proteins. It is therefore important that any future study of dinucleotide-mediated signaling includes a determination of both the synthetic and degradative pathways. A more critical evaluation of Ap_n_N-binding proteins as potentially relevant targets is also required. Because Ap_n_N bind to many ATP-binding proteins, a functional and specific consequence of Ap_n_N binding needs to be demonstrated in addition to binding itself.

The role of Ap_4_A and other Ap_n_N in DNA replication and the DNA damage response also requires clarification. The synthesis of Ap_4_A in response to DNA damage with a resulting inhibition of the initiation of DNA replication may contribute to mechanisms that pause the cell cycle whilst the damage is repaired. Inhibition rather than promotion of replication by Ap_4_A as previously suggested appears to be more plausible. However, there are still many questions. Is the observed inhibition of initiation by Ap_4_A specific for this nucleotide or can other Ap_4_Ns mediate this response and is it due to a priming or competitive inhibition mode of action? Evaluation of inhibition with Ap_3_A, Ap_4_A, Ap_5_A, polyADP-ribosylated-Ap_4_A and Gp_4_G demonstrated that only Ap_4_A could inhibit initiation, but other Ap_n_N have yet to be tested. Given that Ap_4_A can act as a primer for DNA polymerase-α *in vitro*, is it integrated into RNA primers during the initiation phase of DNA replication? If so, would this affect DNA replication kinetics or potentially inhibit this step through primer instability analogous to mRNA decapping? This could perhaps explain the apparent lack of inhibition of the elongation phase of replication by Ap_4_A as leading strand synthesis is possible with fork restart in contrast to initiation and lagging strand synthesis, which require an RNA primer. Finally, recent evidence supporting separate roles for cGAS and STING in the response to endogenous DNA damage raises the possibility that Ap_4_A could be involved in integrating different aspects of the DNA damage response through both the inhibition of DNA replication and endogenous DNA damage sensing. For example, by binding STING, Ap_4_A may promote the assembly of nuclear cGAS-independent STING signaling complexes that lead to NF-κB activation (Unterholzner and Dunphy, 2019). Ap_4_A may therefore be an important player in the mechanisms through which DNA damage activates immune responses to target and eliminate cancer cells.

In conclusion, we suggest that there is now sufficient evidence from both prokaryotes and eukaryotes to demonstrate that dinucleoside polyphosphates have multifaceted signaling activities. We anticipate that there will be significant advances in this area as the Ap_4_N and the Np_n_N family are further characterized, adding to recent observations that have demonstrated their capacity to regulate cellular responses to stress.

## Author Contributions

NC and FF produced the figures. All authors contributed to the writing and editing of the manuscript.

## Conflict of Interest

The authors declare that the research was conducted in the absence of any commercial or financial relationships that could be construed as a potential conflict of interest.
